# Top 100 most-cited articles on renal cell carcinoma: A bibliometric analysis

**DOI:** 10.1097/MD.0000000000032926

**Published:** 2023-02-10

**Authors:** Huiyu Zhou, Fan Cui, Dingyang Lv, Qian Gong, Jie Wen, Weibing Shuang

**Affiliations:** a First Clinical Medical College, Shanxi Medical University, Taiyuan, China; b Department of Urology, The First Hospital of Shanxi Medical University, Taiyuan, China.

**Keywords:** bibliometrics, citation analysis, immunotherapy, renal cell carcinoma, targeted drugs

## Abstract

To analyze the top 100 most-cited articles on renal cell carcinoma (RCC) using bibliometric methods based on the Web of Science core collection database and to explore the research status, hotspots, and emerging trends in RCC.

**Methods:**

The literature on RCC was searched in the Web of Science core collection database using a specific search strategy, and the types of literature were limited to articles and reviews, with no restrictions to language and publication date. The top 100 articles with the highest number of citations were extracted after the manual screening. The publication year, the number of citations, authors, country, institution, journal, and keywords of these articles were collected and analyzed. Descriptive statistics and visual analysis were performed using Microsoft Excel, VOSviewer, CiteSpace, R, and SPSS.

**Results:**

The number of citations of the top 100 articles varied from 541 to 4530, with a median citation count of 807.5, and the citation rates ranged from 13.8 to 448.4 citations per year. Motzer RJ (n = 22), Escudier B (n = 13), Rini BI (n = 13), and Hutson TE (n = 11) were major contributors to this research area, with Motzer RJ publishing 16 articles as the first author. The US (n = 73), France (n = 5), Canada (n = 4), and Sweden (n = 4) were the leading countries for RCC studies. MEMORIAL SLOAN KETTERING CANCER CENTER (n = 22) was the institution with the highest number of publications. These 100 articles were derived from 24 journals, and the *New England Journal of Medicine* had the largest number of articles published (n = 18, impact factor = 91.245). The keyword co-occurrence network analysis showed that research hotspots in this field included molecular mechanisms of RCC development and progression, surgical treatment, targeted drug-related clinical trials, and immunotherapy.

**Conclusion:**

We analyzed the top 100 articles with the highest number of citations in the field of RCC and identified the influential authors, countries, institutions, and journals in this field. This study also presented the current research status, hotspots, and future trends in RCC.

## 1. Introduction

Renal cell cancer (RCC) originates from tubular epithelial cells and accounts for approximately 2 to 3% of adult malignancies.^[1]^ There are about 430,000 new cases and 175,000 deaths each year worldwide.^[2]^ According to pathological classification, there are more than 10 subtypes of RCC, of which 80% belong to clear cell RCC (ccRCC).^[3]^ More than 50% of patients with RCC have no obvious clinical symptoms in the early stages of RCC,^[4]^ so about one-third of patients already have advanced or metastatic disease at the time of initial diagnosis.^[5]^ The prognosis of patients with early RCC is relatively good, with a 5-year overall survival rate of 74%, while the 5-year average survival rate of patients with metastatic RCC is only 8%.^[6]^ Surgical resection is the mainstay of treatment for early-stage localized RCC, though 20 to 30% of patients still relapse or develop metastasis after surgery.^[7]^ In contrast, patients with locally advanced or metastatic RCC do poorly when treated with surgery alone, and they require systemic therapy.^[8]^ In the past few decades, significant progress has been made in the treatment of RCC, especially in the development of systemic therapeutic drugs. The number of relevant publications has also increased remarkably; however, the quality of these articles is uneven, which is difficult for young physician-scientists to identify high-quality research. Therefore, it is necessary to systematically review the literature to identify the high-quality publications in this field.

Bibliometrics is an important tool in scientific research. It can screen and quantitatively analyze the influential literature, determine the hotspots and trends in a certain research field, and provide a unique perspective for researchers and clinicians.^[9,10]^ Among bibliometric methods, citation analysis is the most commonly used one. The analysis of highly cited literature can review the current research status, identify research hotspots, and provide new ideas for future research.^[11]^ Bibliometric methods have been widely used in medical fields, such as dermatology,^[12]^ general surgery,^[13]^ and radiology.^[14]^ To our knowledge, there is still no systematic and comprehensive analysis of the literature on RCC. Therefore, the aim of this study was to analyze the top 100 cited articles in the field of RCC research to better understand the current research status, hotspots, and future directions of RCC.

## 2. Materials and Methods

### 2.1. Data sources and search strategies

An advanced search strategy was used to search the Web of Science core collection database. The time span is 1900 to the present. The search terms included “TS = (renal cell carcinoma) OR TS = (renal cell cancer) OR TS = (kidney cancer) OR TS = (kidney carcinoma) OR TS = (kidney neoplasms) OR TS = (kidney cancer) OR TS = (renal tumor) OR TS = (renal carcinoma).” The types of literature were limited to articles and reviews, with no restrictions to language and publication date. We excluded articles without full text and other complete information, as well as articles studying various cancers including renal cell carcinoma. This study does not involve human subjects, and therefore does not require ethical approval.

### 2.2. Data extraction

The search results were sorted by decreasing frequency of citations. Titles and abstracts for each result were independently screened by 2 professional urologists. When the abstract did not provide the necessary information, the full text was read to exclude articles that were not related to RCC. When there was a disagreement on whether the articles needed to be included, a third urologist was consulted. Finally, the top 100 most cited articles were determined, from which the following information was extracted, including publication year, title, author, country, institution, journal, impact factor (IF) of the journal (2021 edition of Journal Citation Reports), number of citations, citation rate (number of citations divided by the number of years since the publication of the literature), keywords, study type (basic research, clinical research, review, guideline, and consensus), etc.

### 2.3. Data analysis and visualization

Descriptive statistical analysis was performed using Microsoft Excel 2013 (Microsoft Corporation, Redmond, WA). The networks between authors, countries, institutions, and keywords were visualized using VOSviewer 1.6.18.0 software (Leiden University, Leiden, Netherlands). Burst keywords were identified using CiteSpace 5.8 R3 (Drexel University, Philadelphia, PA) to reveal research hotspots. The R package bibliometrix (version 3.1.3) in R (version 4.0.3) was used to draw the views of cooperation networks among various countries around the world.^[15]^ The Spearman correlation coefficient between different parameters was calculated using SPSS 22.0 (IBM Corporation, Armonk, NY), and the correlations are statistically significant at *P* < .05.

## 3. Results

**Figure 1.**
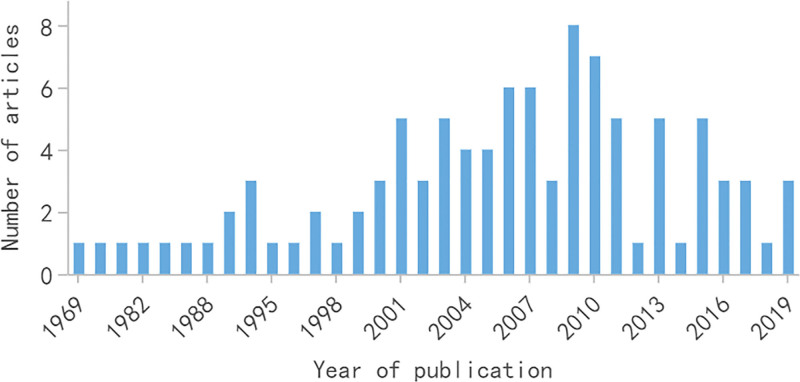
The number of the top 100 most-cited articles on renal cell carcinoma from 1969 to 2017.

A total of 132,712 articles were obtained in this search, and there was an increasing trend in the number of articles over the years. After screening, 100 articles with the highest number of citations were finally determined, all of which were written in English, including 63 clinical studies (30 randomized controlled trials), 23 basic studies, 10 reviews, and 4 guidelines. As shown in Figure 1, these 100 articles were published between 1969 and 2019, and their total citations were 104,937. The total citation per article ranged from 541 to 4530, with a median citation count of 807.5.

The majority (81%) of these top 100 articles were published after 2000, with the largest number of articles published in 2009 (n = 8) and 2010 (n = 7), respectively; and the 5-year period (2006–2010) was the peak period with the highest number of articles published (n = 30). To control for publication time bias, we also calculated citation rates ranging from 13.8 to 448.4 citations per year, with a median of 51.9 citations per year. We found a significant positive correlation between citation rate and publication year (*R* = 0.728, *P* < .001). The number of citations that the top 10 articles with the highest number of citations cited accounted for 25.4% of the total number of citations (Table 1). Six articles of the top 10 articles with the highest citation rates were still among the top 10 articles with the highest number of citations, but the remaining 4 articles published in the last 10 years were not included due to they had not enough time to accumulate citations. It is worth noting that 9 of the top 10 cited articles focused on randomized controlled trials on targeted immunotherapy in RCC.

**Table 1:** Top 10 most-cited articles on renal cell carcinoma.

Rank	Title	First author	Citation	Year	Average per yr (rank)
1	Sunitinib versus interferon alfa in metastatic renal-cell carcinoma	Motzer, RJ	4530	2007	283.1(4)
2	Sorafenib in advanced clear-cell renal-cell carcinoma	Escudier, B	3883	2007	242.7 (6)
3	Nivolumab versus everolimus in advanced renal-cell carcinoma	Motzer, RJ	3587	2015	448.4 (1)
4	Temsirolimus, interferon alfa, or both for advanced renal-cell carcinoma	Hudes, G	2973	2007	185.8 (8)
5	Efficacy of everolimus in advanced renal cell carcinoma: a double-blind, randomized, placebo-controlled phase III trial	Motzer, RJ	2382	2008	158.8 (10)
6	Prognostic significance of morphologic parameters in renal cell carcinoma	Fuhrman, SA	2177	1982	53.1 (50)
7	A randomized trial of bevacizumab, an anti-vascular endothelial growth factor antibody, for metastatic renal cancer	Yang, JC	2168	2003	108.4 (19)
8	Nivolumab plus ipilimumab versus sunitinib in advanced renal-cell carcinoma	Motzer, RJ	1954	2018	390.8 (2)
9	Pazopanib in locally advanced or metastatic renal cell carcinoma: results of a randomized phase III trial	Sternberg, CN	1859	2010	143 (12)
10	Bevacizumab plus interferon alfa-2a for treatment of metastatic renal cell carcinoma: a randomized, double-blind phase III trial	Escudier, B	1841	2007	115.06 (17)

### 3.1. Authors

**Figure 2.**
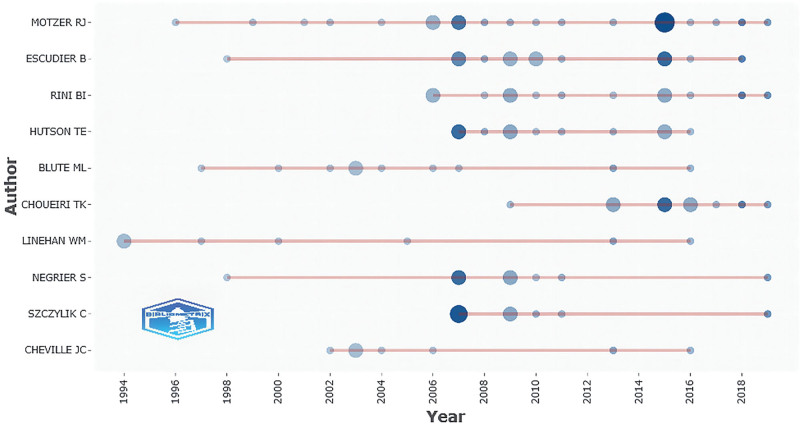
Top 10 authors contributed to the top 100 most-cited articles.

**Figure 3.**
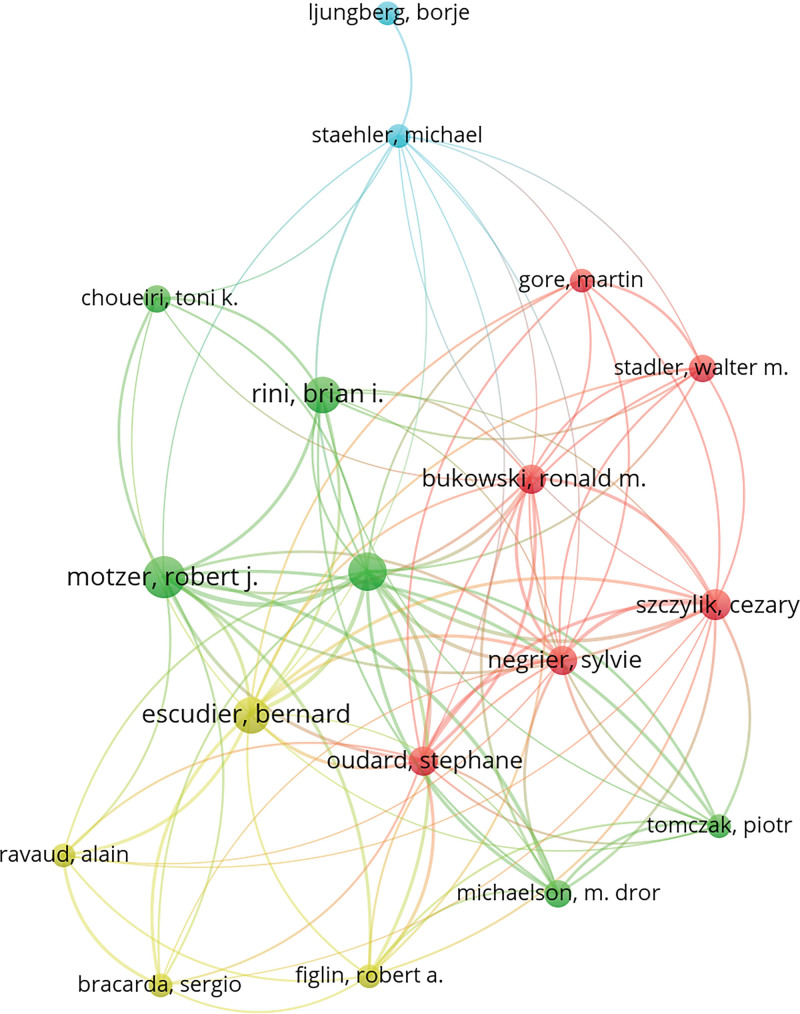
Visualization of author collaboration network. The size of nodes indicates the number of authors who published the top 100 most-cited articles. The connection between nodes indicates the collaborative relationship between authors. Different colors of nodes and connection represent the collaboration between different authors.

A total of 1281 authors participated in these 100 articles, and the top 10 authors with the most contributions are listed in Table 2. Motzer RJ (n = 22), Escudier B (n = 13), Rini BI (n = 13), and Hutson TE (n = 11) were major contributors to this research area. Of these 10 authors, 7 were from the USA, 2 from France, and 1 from Poland. A total of 11 authors contributed >1 article as the first author, of which Motzer RJ published 16 articles as the first author, followed by Rini BI (n = 5) and Escudier B (n = 4). Although both Hutson TE and Blute ML contributed to at least 10 articles, they were not listed as the first author. As shown in Figure 2, Motzer RJ and Linehan WM spent the longest time studying RCC, and Motzer RJ published high-quality articles in 2015. In addition, there was a collaboration network among 18 authors who contributed to ≥4 articles (Fig. 3). The network was led by Motzer RJ, Escudier B, Rini BI, and Hutson TE. Hutson TE, Motzer RJ, Escudier B, Szczylik C, and Negrier S collaborated closely with other authors, suggesting that they have a closer partnership with others.

**Table 2:** Top 10 authors contributing to the top 100 most-cited articles.

Rank	Author	Total number of articles	Number of articles as first author	Country
1	Motzer RJ	22	16	USA
2	Escudier B	13	4	France
3	Rini BI	13	5	USA
4	Hutson TE	11	0	USA
5	Blute ML	10	0	USA
6	Choueiri TK	10	3	USA
7	Negrier S	8	0	France
8	Szczylik C	8	0	Poland
9	Cheville JC	7	1	USA
10	Linehan WM	7	1	USA

### 3.2. Country

**Figure 4.**
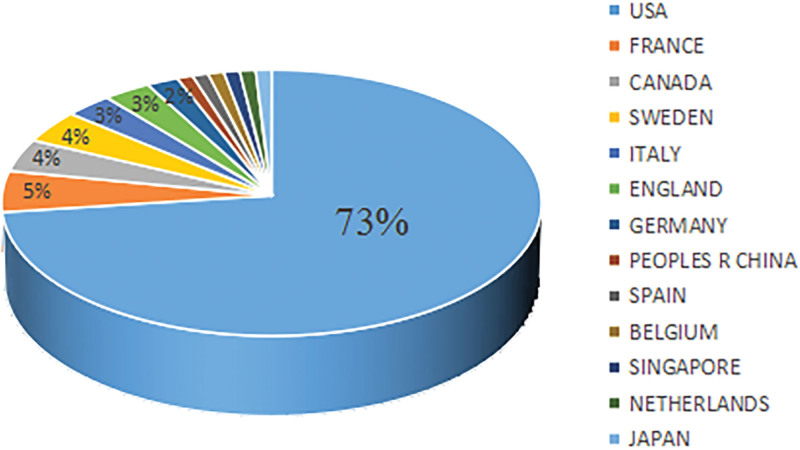
Countries of origin publishing the top 100 most-cited articles.

**Figure 5.**
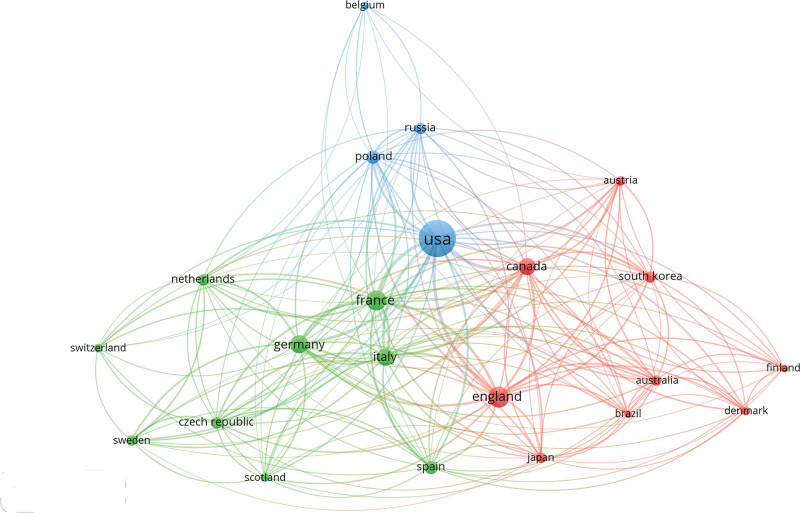
The international collaboration network among countries. The size of nodes indicates the number of the top100 most-cited articles published by different countries. The connection between nodes indicates the collaborative relationship between countries. Different colors of nodes and connection represent the collaboration between different countries.

**Figure 6.**
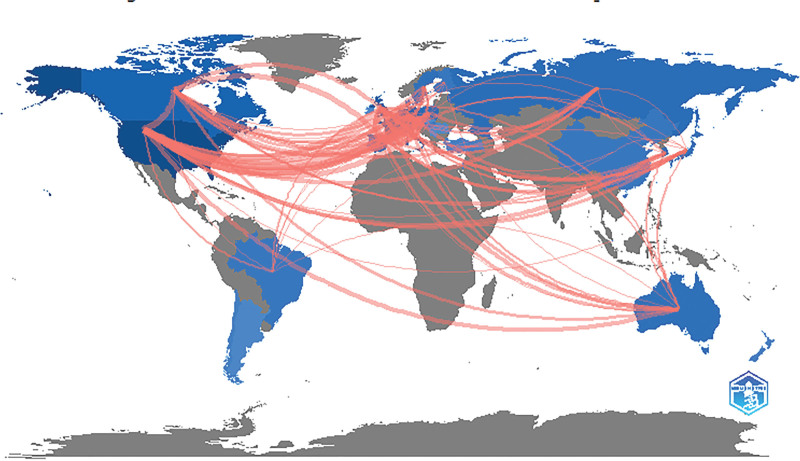
Visualization of the collaboration network of countries around the world. The depth of color indicates the number of the top100 most-cited articles. The connection represents the collaboration between countries. The width of lines represents the number of coauthored articles.

Based on the country of the first author, the top 100 cited articles were published by 13 countries, which included 12 developed countries and 1 developing country. As shown in Figure 4, the US published the highest number of articles (n = 73), followed by France (n = 5), Canada (n = 4), and Sweden (n = 4). An international collaboration network comprising the countries that contributed to ≥4 articles was formed (Fig. 5). The US, France, and the UK have close cooperation with other countries. At the continental level, North America and Europe published the largest number of articles (Fig. 6). The authors from North America actively collaborated with those from Europe, Asia, and Oceania. However, none of the 100 cited articles were published by researchers in Africa, indicating the presence of a scientific output gap between developing and developed countries.

### 3.3. Institution

**Figure 7.**
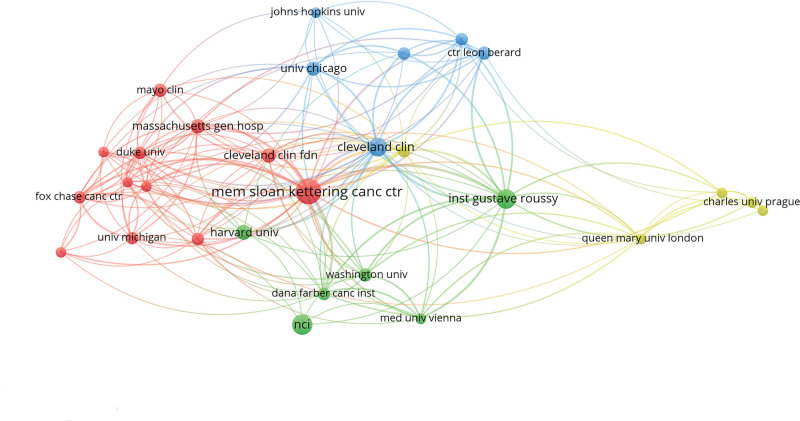
The collaboration network among different institutions. The size of nodes indicates the number of articles published by institutions. The connection between nodes indicates the collaborative relationship between institutions. Different colors of nodes and connection represent the collaboration between different institutions.

A total of 366 institutions contributed to the publication of the 100 most cited articles (Table 3). MEMORIAL SLOAN KETTERING CANCER CENTER published the highest number of papers (n = 22) because of the affiliation of Motzer RJ with this institution, which was followed by NCI (n = 14) and INST GUSTAVE ROUSSY (n = 13). MEMORIAL SLOAN KETTERING CANCER CENTER also had the highest number of citations, indicating that this institution has been well-recognized by peers due to its high-quality publications. In addition, MEMORIAL SLOAN KETTERING CANCER CENTER, INST GUSTAVE ROUSSY, and CLEVLAND CLIN collaborated closely with other institutions (Fig. 7).

**Table 3:** Top 5 institutions contributing to the top 100 most-cited articles.

Rank	Institution	Number	Citation	Country
1	MEMORIAL SLOAN KETTERING CANCER CENTER	22	28061	USA
2	NCI	14	13786	USA
3	INST GUSTAVE ROUSSY	13	16894	France
4	CLEVLAND CLIN	12	14038	USA
5	HARVARD UNIV	8	7462	USA

### 3.4. Journal

**Figure 8.**
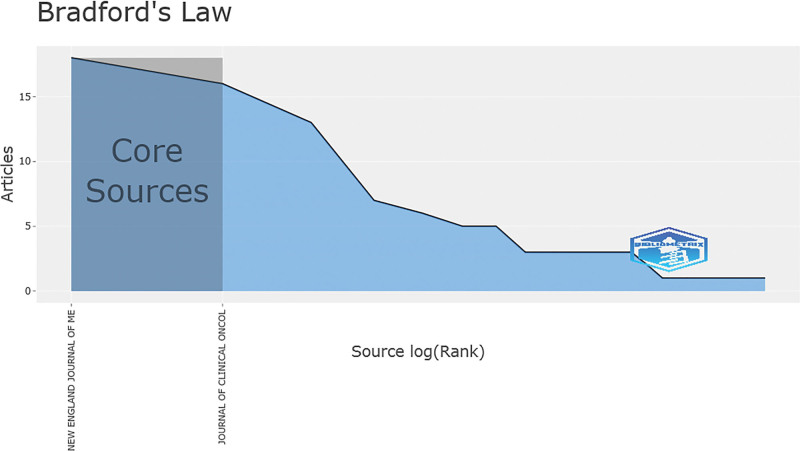
Core journals in which the top100 most-cited articles were published.

These 100 articles were published in 24 journals, and the top 10 journals with the highest number of publications were shown in Table 4. *New England Journal of Medicine* published the highest number of articles (n = 18, IF = 91.245), followed by the *Journal of Clinical Oncology* (n = 16, IF = 44.544) and *Journal of Urology* (n = 13, IF = 7.450). *New England Journal of Medicine* also had the highest number of citations (1695.56 times/article), indicating that this journal has been recognized as the world’s leading medical journal. Among these 10 journals, 6 were published in the US, 3 in the UK, and 1 in the Netherlands. The average IF of these 24 journals was 35.69 (range: 2.241–91.245), indicating that the academic influence of RCC research was high. In addition, there was a positive correlation between journal IF and the number of articles (*R* = 0.433, *P* = .034), the total number of citations (*R* = 0.511, *P* = .011), and the number of citations for all articles (*R* = 0.460, *P* = .024). According to Bradford’s Law, *New England Journal of Medicine* and *Journal of Clinical Oncology* belonged to the core journals (Fig. 8).

**Table 4:** Top 10 journals with the highest number of top most-cited articles.

Rank	Journal	Number of articles	IF (2021)	Citations/Numbers	Country
1	*New England Journal of Medicine*	18	91.245	1695.56	USA
2	*Journal of Clinical Oncology*	16	44.544	1012.25	USA
3	*Journal of Urology*	13	7.450	828.54	USA
4	*European Urology*	7	20.096	832.14	Netherlands
5	*Nature Genetics*	6	38.33	924.17	England
6	*Lancet*	5	79.321	1528.2	England
7	*Nature*	5	49.962	936.8	England
8	*American Journal of Surgical Pathology*	3	6.394	1213	USA
9	*Cancer*	3	6.86	834.67	USA
10	*Cancer Research*	3	12.701	632.67	USA

IF = impact factor.

### 3.5. Visualization of keyword co-occurrence network

**Figure 9.**
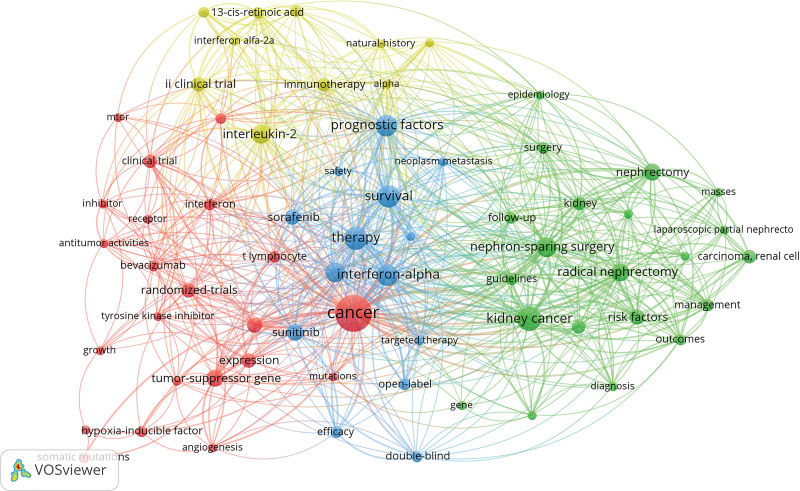
Clustering analysis of keyword co-occurrences. The size of nodes indicates the number of keyword co-occurrences. The line between 2 nodes indicates the co-occurrences of keywords. Each color represents one cluster of keywords.

**Figure 10.**
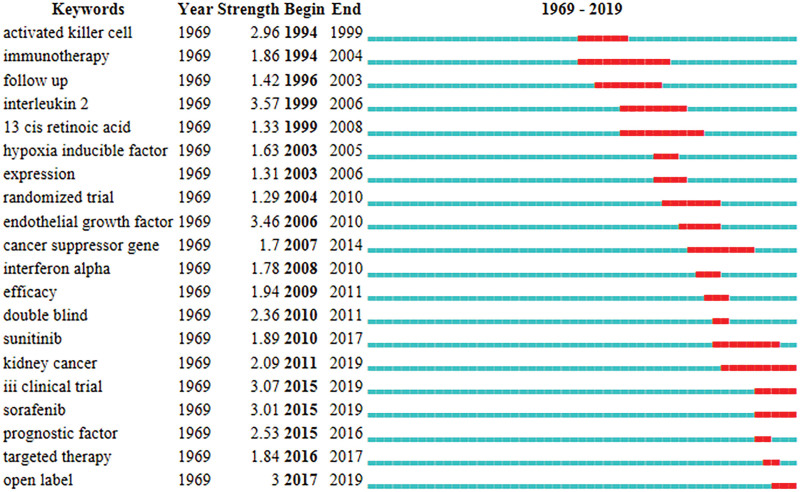
Top 20 keywords with strongest citation bursts in the top 100 most-cited articles. Blue lines indicate the timeline during which they are rarely cited, and red lines indicate the timeline during which citation bursts occur. The strength value represents the frequency of citation.

High-frequency keywords can accurately reveal hotspots in a research field.^[16]^ Before performing the analysis, we first manually merged the same keywords with different expressions, and then identified a total of 386 keywords, of which 255 keywords appeared only once. Table 5 lists the top 10 keywords with the highest number of occurrences. The visualization of keyword co-occurrence network was performed using VOSviewer to identify research hotspots of RCC more intuitively and quickly. As shown in Figure 9, a total of 67 keywords appeared ≥3 times in the top 100 cited articles, and they were divided into 4 clusters. The largest cluster (red) contained 22 keywords, such as cancer, endothelial growth factor, a tumor-suppressor gene, expression, etc. The second largest cluster (green) consisted of 21 keywords, including kidney cancer, nephron-sparing surgery, radical nephrectomy, nephrectomy, surgery, epidemiology, and risk factors. The third cluster (blue) consisted of 14 keywords, including therapy, survival, interferon-α, III clinical trials, prognostic factors, open-label, etc. The fourth cluster (yellow) was the smallest one and contained 10 keywords, including interleukin-2, immunotherapy, α, and tumor-infiltrating lymphocytes. The visualization of these high-frequency keywords showed that the research hotspots were mainly concentrated on the treatment of RCC. We identified twenty keywords with the highest citation bursts using Citespace. It can be seen that in systemic therapy for RCC, the research hotspots have shifted from traditional immunotherapy (such as interferon α and interleukin-2) to targeted drugs (such as sunitinib and sorafenib) in recent years, and the major type of research was an open-label phase III clinical trial (Fig. 10).

**Table 5:** Top 10 keywords with the highest number of occurrences in the top 100 most-cited articles.

Rank	Keywords	Number	Total link strength
1	Cancer	45	200
2	Kidney cancer	19	97
3	Therapy	18	99
4	Survival	17	94
5	Prognostic factors	15	78
6	Interferon-α	15	76
7	Nephron-sparing surgery	13	84
8	III clinical trial	13	80
9	Interleukin-2	12	73
10	Radical nephrectomy	11	70

## 4. Discussion

For the first time, we identified 100 top-cited articles in the field of RCC using bibliometric methods and revealed the status, hotspots, and trends in the research of RCC. This work is not only important for researchers to select future research fields, but also helps governments and institutions to reasonably allocate limited health resources. A highly cited article is regarded as a milestone that can have a considerable impact on future research and clinical decision-making.^[17]^ In general, articles with 400 citations can be regarded as “classical citations.”^[18]^ It is worth mentioning that the 100 articles identified in this study were “classical citations,” of which 34 articles had more than 1000 citations. The publication of a large number of high-quality articles indicates that there is a huge research space in the field of RCC.

The earliest most-cited article was published in 1969, and the most recent most-cited article was published in 2019. There were 81 most-cited articles since 2000, indicating that there has been a breakthrough in the field of RCC and the research interest in RCC has increased remarkably over the past 20 years. It should be noted that, besides the quality of the articles, the number of citations is closely related to their publication time. We used citation rates to reduce the impact of the post-publication period on the number of citations of articles published earlier. Articles with both a high number of citations and a high citation rate can reflect the current research status and hotspots. In contrast, articles with a high number of citations but a low citation rate are more historically important. In general, the scientific literature is usually cited 1 or 2 years after publication and reaches the peak of citation about 10 years after publication.^[19]^ Therefore, although the literature published in the last 2 years was not included in this study, their influence should not be underestimated. We found a significant positive correlation between the citation rate and the publication year (*R* = 0.728, *P* < .001). It might be related that people prefer to cite recent articles. In contrast, the findings from the early most-cited articles are so universally accepted, and their source is often forgotten, resulting in a reduced citation rate.^[19]^ For example, the article written by Fuhrman SA et al in 1982 was cited 2177 times and ranked the sixth, but its citation rate was only 53.1 times/year and ranked the 50th. The authors evaluated the prognostic significance of morphological parameters of tumors in 103 patients with RCC and found that nuclear grading was valuable in predicting the long-term outcomes of patients and could be applicable to various tumors.^[20]^ Afterward, the Fuhrman nuclear grading system was developed and widely used in the grading of RCC. However, the Fuhrman grading system has been integrated into the latest criteria for prognostic evaluation of RCC, such as the Mayo Clinic Stage, Size, Grade, and Necrosis scoring system^[21]^ and the University of California Los Angeles integrated staging system,^[22]^ leading to a reduced citation rate. The article with the highest citation rate of 448.4 citations/year was published by Motzer RJ in 2015, in which the authors conducted a randomized, open phase III clinical trial and found that nivolumab was more effective than everolimus in treating patients with advanced RCC.^[23]^ The article with the largest number of citations was published by Motzer RJ in 2007, in which the authors conducted a multicenter, randomized phase III trial and found that sunitinib increased progression-free survival and response rates in patients with metastatic RCC compared to interferon alfa.^[24]^

Of 1281 authors who published the top 100 most-cited articles, 76.50% appeared only once, indicating that only a few authors have been continuously engaged in RCC research and published several influential articles. The author collaboration network showed the collaborative relationship among these authors (Fig. 3). It can be seen that Hutson TE, Motzer RJ, Escudier B, Szczylik C, and Negrier S collaborated actively with other researchers. Three authors, Motzer RJ, Escudier B, and Rini BI contributed to 34% of the 100 top-cited articles and nearly 27% of citations. Motzer RJ in the US was a leading author in the long-term research of RCC. Like in other medical fields (e.g., ophthalmology, dermatology), the authors from the US played a dominating role in the research field of RCC and published two-thirds of the 100 top-cited articles.^[13,25,26]^ In addition, the authors from the US built effective partnerships with researchers from different countries in scientific research and kept a good scientific reputation. There was only one developing country among 13 countries that published the 100 top-cited articles. Therefore, developing countries needed to strengthen their cooperation with developed countries to improve their scientific research. In terms of geographical distribution, Europe and North America published the majority of the 100 top-cited articles, while Asia and South America contributed little to these publications. Besides economic factors, this phenomenon might also be related to the high incidence of RCC in Europe and North America.^[27]^ MEMORIAL SLOAN KETTERING CANCER CENTER in the US published the largest number of top-cited articles on RCC (n = 22), which is related to the dedication of Motzer RJ to RCC research and the high-quality research output of this institution. During the era of globalization, it is necessary to strengthen international cooperation between developed and developing countries in science and technology.

The 100 top-cited articles were published in 24 journals, 16 of which originated in the USA. It is reported that authors in the USA usually prefer to publish their research in US-based journals and to cite the literature published in the USA.^[28]^ The number of citations is not only an indicator of the impact of an article in the scientific community but also the basis for calculating the IF of a journal.^[11]^ Spearman correlation analysis showed that there was a positive correlation between the journal’s IF and the number of articles, the total number of citations, and the number of citations, indicating that journals with high IF attracted more high-quality articles, and researchers were more willing to read and cite articles published in high-impact journals, which in turn could further improve the academic reputation of these journals.^[29]^ According to Bradford’s Law proposed by Brookes BC, most researchers receive more citations from high-impact journals in their respective professional fields, and the impact of their articles and the number of citations decrease when the articles are not published in these journals.^[30]^ Our work also supported this law and found that the *New England Journal of Medicine* and *Journal Of Clinical Oncology* were journals in which over one-third of the top-cited articles were published.

Second, we found that clinical research (especially randomized controlled trials) accounted for a large proportion of the top-cited articles compared with basic research and reviews, possibly due to clinicians’ preference to publish clinical data rather than the results of basic research. In general, randomized controlled trials are considered the highest level of evidence in medical research^[31]^ and are an important part of clinical research.^[32,33]^ Randomized controlled trials are gaining popularity because the development of reliable guidelines requires a high level of evidence-based research. This trend also reflects the concept of evidence-based medicine. However, there are many challenges facing randomized controlled trials, such as multi-center collaboration, a large number of personnel, and huge funding gaps. Collaboration among different countries, institutions, and authors is key for the implementation of large-scale clinical trials. Among the top 10 cited articles, Motzer RJ conducted 4 large randomized controlled trials in the US, including sunitinib versus Interferon Alfa (750 patients with previously untreated metastatic RCC),^[24]^ nivolumab versus Everolimus (821 patients with previously treated advanced RCC),^[23]^ everolimus versus placebo (410 patients with previously treated metastatic RCC),^[34]^ and nivolumab + ipilimumab versus sunitinib (1096 patients with previously untreated advanced RCC),^[35]^ all of which enrolled patients with ccRCC. Thus, Motzer RJ has made great contributions to clinical trials of drugs to treat RCC. Systematic reviews and meta-analyses aimed to assess the quality of clinical trials to provide the highest level of evidence. However, these 2 types of articles were not found in the top 100 most-cited articles. There were 23 articles that presented the results of basic research and focused on biomarkers and gene mutations in RCC. The proportion of reviews in the literature is not very high, but they play an important role in collecting information in the field.^[36]^ Guidelines and consensus are the reevaluation and integration of relevant evidence, providing the current evidence base for the management of RCC.

Keywords represent the main concepts of research topics in science and technology. The cluster analysis of keyword co-occurrences can visualize the correlation between keywords, which can help researchers identify current research hotspots and emerging trends. The keywords in the largest clusters (red) are mainly associated with the exploration of molecular mechanisms underlying the development and progression of RCC, which lay the foundation for the discovery of potential prognostic biomarkers and targeted therapeutics in RCC. The keywords in the second largest clusters (green) are mainly related to surgical treatment, epidemiology, and risk factors. Since the first reported case of laparoscopic nephrectomy by Clayman RV et al in 1991,^[37]^ laparoscopy has been widely used in surgery. Interestingly, the case report was one of the top 100 most-cited articles, with a total of 1089 citations and a citation rate of 30.0 citations/year. Compared to traditional open surgery, laparoscopic and robot-assisted nephrectomy have been increasingly recommended by clinicians.^[38]^ Partial nephrectomy is the first-line treatment for localized RCC. Recently, the R.E.N.A.L. nephrometric score has been used to assess the complexity and feasibility of partial nephrectomy to surgically treat RCC based on imaging findings, thereby reducing the risk of cardiovascular disease.^[39,40]^ Obesity, hypertension, and smoking are risk factors for RCC.^[27]^ We found that a decline in the incidence of RCC in developed countries could be attributed to changes in lifestyle, especially reduced smoking and adoption of healthy lifestyles.^[41]^ The keywords in the third largest cluster (blue) were mainly associated with clinical trials of targeted drugs, such as sunitinib, sorafenib, etc. The keywords in the smallest cluster (yellow) are mainly related to immunotherapy in RCC. With the in-depth understanding of immune escape mechanisms and the development of new technologies (such as sequencing), a variety of novel immunotherapies with high efficacy have been developed. The keyword burst analysis suggests that targeted drugs and open clinical trials have become current research hotspots and trends in RCC.

Since the 1970s, cytokine therapy has been widely used in the treatment of advanced RCC, however, it has some shortcomings, such as low response rates and high toxicity.^[42,43]^ Since 2005, a variety of targeted and novel immunotherapeutic agents have been approved for the treatment of patients with locally advanced and metastatic RCC, which prolong the progression-free survival and overall survival of patients. At the same time, more and more clinical trials have also been conducted to compare the efficacy and safety of different drugs. Inactivation of the von Hippel–Lindau tumor suppressor gene is found in patients with hereditary RCC and 80% of patients with sporadic ccRCC, and the inactivation resulted in the accumulation of hypoxia-inducible factor-1 and high expression of vascular endothelial growth factor (VEGF) and platelet-derived growth factor, ultimately leading to tumor growth and metastasis.^[44–46]^ Mammalian target of rapamycin (mTOR) is a protein kinase that regulates cell metabolism, growth, and proliferation and is closely related to the development of RCC.^[47]^ Understanding the signaling pathways involved in RCC can enhance the development of molecularly targeted therapy. At present, the commonly used targeted drugs in clinical practice are mainly divided into 2 categories: VEGF pathway inhibitors and mTOR inhibitors. The VEGF pathway inhibitors include sorafenib, sunitinib, bevacizumab, pazopanib, and axitinib, which mainly prevent tumor growth and invasion by inhibiting tumor angiogenesis. The mTOR inhibitors include everolimus and temsirolimus, which mainly inhibit the proliferation and division of tumor cells and promote their apoptosis by interfering with signal transduction pathways.^[48]^ These drugs have been approved by the FDA and have shown good results in clinical trials. Since cabozantinib and lenvatinib have only recently been approved, there are few relevant studies on them. The National Comprehensive Cancer Network and European Association of Urology recommend sunitinib and pazopanib as first-line regimens for patients with low-risk advanced ccRCC and recommend axitinib and cabozantinib as second-line options for patients with advanced ccRCC. The mTOR inhibitors (everolimus and temsirolimus) are approved as single agents for second-line and first-line treatment of patients with low-risk metastatic RCC.^[34,49]^ Although targeted drugs have greatly improved the survival of patients with advanced and metastatic RCC, some patients have experienced drug resistance and tumor progression.^[50]^ Studies have shown that 30% of patients with metastatic ccRCC have primary resistance to molecularly targeted drugs, and some patients develop secondary resistance after 1 year of treatment, ultimately leading to a poor prognosis.^[51]^ However, the specific mechanisms of drug resistance are still poorly understood, so it should be noted that elucidating the resistance mechanisms of targeted drugs and how to combine them are future research priorities.

With the in-depth understanding of immune infiltration, immune escape, and tumor microenvironment,^[52]^ a number of new immunotherapies have emerged in recent years. Immune checkpoint inhibitors (ICIs) can promote the immune-mediated killing of tumor cells by activating T cells,^[53]^ and these ICIs include anti-programmed cell death-1 (nivolumab and pembrolizumab), anti-programmed cell death ligand-1 (bavencio and tecentriq), and anti-cytotoxic T-lymphocyte antigen-4 (ipilimumab). Nivolumab is the first ICIs that was officially approved in the US and the European Union in 2015 for the second-line treatment of advanced RCC. Targeted drugs not only have an anti-angiogenic effect but also can induce tumor microenvironment. For example, sunitinib can reverse the inhibitory effect of advanced RCC tumors on T cells,^[54]^ suggesting that ICIs and anti-angiogenic drugs may have a synergistic effect. Therefore, the current trend in the treatment of RCC is combination therapy strategies, such as the combination of 2 ICIs, the combination of anti-angiogenic drugs, and ICIs.^[55]^ Nivolumab plus ipilimumab combination therapy or axitinib combined with pembrolizumab has been approved in the US and Europe for the first-line treatment of intermediate- and low-risk advanced or metastatic RCC.^[56]^ The combination of immune-targeted drugs will certainly open a new era for the treatment of RCC, and the development of new technologies (such as gene sequencing) is helpful for the development of individualized treatment plans for patients with RCC.

In addition, our study has several limitations. First, the literature used for bibliometric analysis was extracted only from the Web of Science, which might neglect some highly cited articles included in some other databases (such as SCOPUS, PubMed, and Google Scholar). The analysis of these databases might show different results.^[57]^ Nevertheless, the Web of Science is still the most widely used database in bibliometric studies at present.^[58]^ Second, the number of citations is only an indicator of the academic influence of the literature and does not fully reflect the quality of the literature. Various factors, such as publication time, research area, and specialty, may affect the number of citations, leading to discrepant citations of references.^[59]^ For example, authors who continue to conduct research in a certain subfield may cite their own previously published papers, or some authors prefer to cite articles published in the journals to which they want to submit their manuscripts.^[60]^ Third, high-impact articles published in recent years are not included in this study because they do not have enough time to accumulate citations. Fourth, the top 100 most-cited articles might vary with the changes in research hotspots, so they need to be continuously updated in the future. Despite these limitations, this descriptive bibliometric study can still provide unique insights into the current research status and emerging trends in RCC.

In this study, we analyzed the top 100 most-cited articles on RCC and identified high-impact authors, countries, institutions, and journals in this field. This study can provide researchers and clinicians with the current research status, hotspots, and emerging trends in RCC. In addition, this work can serve as guidance for R & D planning and funding decisions.

## Author contributions

**Conceptualization:** Huiyu Zhou.

**Data curation:** Huiyu Zhou.

**Formal analysis:** Fan Cui.

**Methodology:** Fan Cui.

**Software:** Dingyang Lv, Qian Gong.

**Supervision:** Weibing Shuang.

**Validation:** Fan Cui, Qian Gong.

**Writing – original draft:** Huiyu Zhou.

**Writing – review & editing:** Dingyang Lv, Jie Wen, Weibing Shuang.
